# SunGold Kiwifruit Consumption Restores Adequate to Optimal Vitamin C Status in People with a History of Severe Respiratory Infections

**DOI:** 10.3390/antiox13030272

**Published:** 2024-02-23

**Authors:** Emma Vlasiuk, Masuma Zawari, Malina Storer, Michael J. Maze, Jonathan Williman, Stephen T. Chambers, Anitra C. Carr

**Affiliations:** 1 Nutrition in Medicine Research Group, Department of Pathology and Biomedical Science, University of Otago, Christchurch 8011, New Zealand; emma.vlasiuk@otago.ac.nz (E.V.); masuma.zawari@otago.ac.nz (M.Z.); 2 Respiratory Services, Christchurch Hospital, Christchurch 4710, New Zealand; malina.storer@cdhb.health.nz; 3 Department of Medicine, University of Otago, Christchurch 8011, New Zealand; michael.maze@otago.ac.nz; 4 Department of Population Health, University of Otago, Christchurch 8011, New Zealand; jonathan.williman@otago.ac.nz; 5 The Infection Group, Department of Pathology and Biomedical Science, University of Otago, Christchurch 8011, New Zealand; steve.chambers@otago.ac.nz

**Keywords:** respiratory infections, kiwifruit, vitamin C, inflammation, oxidative stress, fatigue, mood, respiratory symptoms

## Abstract

Severe respiratory infections are characterised by depleted vitamin C and elevated inflammation and oxidative stress. The aim of this study was to recruit people with a history of severe respiratory infections to undergo a six-week intervention with SunGold kiwifruit to determine if this could restore adequate vitamin C status. Secondary outcomes included changes in inflammatory and oxidative stress biomarkers, self-reported fatigue and subjective mood, and the incidence, duration and severity of respiratory symptoms. The total cohort comprised 20 adults (65% female, age range 31–84 years). The participants had a low median fruit and vegetable intake of 2.3 servings/day and a correspondingly low vitamin C intake of 46 mg/day. Circulating vitamin C status was a median of 45 µmol/L and was in the hypovitaminosis range in 25% of the cohort. Following intervention with two SunGold kiwifruit/day (equivalent to ~300 mg vitamin C), there was an increase in plasma vitamin C concentrations to >60 µmol/L (*p* < 0.05). Approximately 20% of the participants were unable to reach adequate vitamin C status (≥50 µmol/L), possibly due to current smoking, which enhances vitamin C turnover, and a strong inverse correlation between body weight and vitamin C status (r = −0.734, *p* < 0.05). Following the intervention, there were indications towards decreases in the inflammatory biomarkers C-reactive protein and TNFα (*p* > 0.05), but no changes in oxidative stress biomarkers (F_2_isoprostanes, protein carbonyls). There were decreases in fatigue and depression (*p* < 0.05) and a lower number of individual respiratory symptoms reported during the kiwifruit intervention phase (8.5 vs. 10, *p* = 0.05). Overall, the consumption of two SunGold kiwifruit per day for six weeks was able to restore adequate to saturating vitamin C status in ~80% of the participants. Smokers and people with higher body weight may need larger doses and/or longer duration of supplementation. The contribution of vitamin C to reducing fatigue, depression, and number of respiratory symptoms warrants further investigation.

## 1. Introduction

Vitamin C, an essential dietary nutrient that must be obtained via our diet, is known to support both the innate and adaptive immune systems [1]. It is noteworthy that severe respiratory infections, such as pneumonia, are one of the most common complications of prolonged vitamin C deficiency, indicating a strong connection between vitamin C insufficiency and respiratory infections [2]. Patients with severe respiratory infections have depleted vitamin C status, which may be both a contributor to and a consequence of the inflammatory process [3,4,5,6]. This depletion can occur even when receiving recommended nutrient intakes [7,8], indicating that patients with severe infections have enhanced turnover and requirements for vitamin C [9,10].

People who experience a severe respiratory infection or those with underlying lung disease who experience recurrent infections can experience a vicious cycle which consists of airway infection, inflammation, and structural damage, which facilitates further infection [11,12,13]. Respiratory infections are characterised by elevated inflammation, including potential ‘cytokine storm’ in severe cases [14], as well as oxidative stress, comprising an imbalance in the generation of reactive oxygen species in the body and the ability of endogenous antioxidants to combat this [15,16]. Vitamin C is a potent antioxidant that can scavenge a wide range of reactive oxygen species [17], thus protecting host tissue from oxidative damage and dysfunction and thereby potentially improving patient outcomes. In confirmation of this premise, intervention studies in people with severe respiratory infections have demonstrated improved vitamin C status as well as decreased respiratory symptoms and decreased duration of hospital stay [4,5].

Kiwifruit are an excellent food source of vitamin C [18]. Vitamin C uptake from kiwifruit is comparable to tablets [19], and their value as a food source is the presence of additional micronutrients and phytochemicals that may provide added health benefits. A previous prophylactic crossover study comprising 32 elderly people supplemented with the equivalent of four gold kiwifruit daily for four weeks showed a decrease in the duration and severity of selected symptoms of upper respiratory tract infections [20]. Thus, both vitamin C supplementation and vitamin C-rich kiwifruit consumption appear to improve selected respiratory tract symptoms in a therapeutic and prophylactic manner [4,5,20]. 

The aim of our study was to determine if a six-week intervention with SunGold kiwifruit (two per day, equivalent to ~300 mg of vitamin C) could restore adequate vitamin C status in people with a history of severe respiratory infections. Secondary outcomes included changes in inflammatory and oxidative stress biomarkers, self-reported fatigue and subjective mood, and the incidence, duration and severity of common respiratory symptoms. We hypothesised that the supplementation of kiwifruit to these people would enhance their vitamin C status and may decrease inflammatory and oxidative stress biomarkers, as well as improve fatigue, subjective mood, and respiratory symptoms. 

## 2. Materials and Methods

### 2.1. Study Design

This was a single-arm ‘before and after’ study designed to assess the impact of six-week SunGold kiwifruit supplementation on vitamin C status in people with a history of severe respiratory infections. The trial received ethical approval from the New Zealand Northern B Health and Disability Ethics Committee (#21/NTB/138, 31 May 2021) and was registered with the Australian New Zealand Clinical Trials Registry (https://www.anzctr.org.au, #ACTRN12621000769886, 21 June 2021). All participants provided written informed consent to participate in the study, which was conducted according to the principles of the Declaration of Helsinki at the University of Otago, Christchurch, New Zealand, with rolling recruitment occurring between July and December 2022 and between March and October 2023.

The primary outcome was the proportion of people reaching adequate plasma vitamin C status following six weeks of intervention. Secondary outcomes included changes in inflammatory biomarkers (C-reactive protein, CRP; interleukin-6, IL-6; tumour necrosis factor-alpha, and TNFα), oxidative stress biomarkers (F_2_isoprostanes and protein carbonyls), self-reported fatigue, and subjective mood (anxiety and depression). The incidence, duration, and severity of respiratory symptoms were also assessed during the intervention period and compared with a subsequent six-week follow-up period which took place after a four-week washout period.

### 2.2. Participant Recruitment and Eligibility Criteria

We recruited people with previous hospitalisation for severe respiratory infection requiring intravenous (IV) antimicrobial therapy. Inclusion criteria were adults aged ≥18 years who had IV antimicrobial therapy administered for pneumonia or an infective exacerbation of underlying respiratory disease within the previous 12–18 months. Exclusion criteria included not being able to give consent, severe coexisting medical conditions that would worsen the prognosis (e.g., heart failure, lung cancer, or cystic fibrosis), a known kiwifruit allergy, and taking vitamin C-containing supplements at greater than the NZ recommended dietary intake (i.e., >45 mg/day). Potential participants were identified from Christchurch Hospital clinical records and were contacted by the study coordinator.

A sample size of 13 would provide 90% power at a two-sided alpha of 0.05 to detect a change in mean vitamin C concentrations from 47 µmol/L to 57 µmol/L [21]. This assumes a within-subject standard deviation of 10 µmol/L or an effect size of 1. This equates to an absolute change of 35% in the proportion of patients achieving adequate levels of vitamin C, i.e., ≥50 μmol/L (from 40% pre-intervention to 75% or more post-intervention). Since pre-screening for inadequate vitamin C status (i.e., <50 µmol/L) was not carried out as part of the study inclusion criteria, we recruited additional participants to account for this (*n* = 20 total). 

### 2.3. Baseline Clinical Data

Participant data were collected and managed using REDCap (Research Electronic Data Capture; Version 13.10.0), a secure, web-based data collection and storage tool hosted by the University of Otago. At baseline, clinical data, demographics (age, gender, and ethnicity) and socioeconomic status were collected at the time of clinic visit and from clinical notes (Health Connect South) and entered into the REDCap database. Clinical data comprised parameters routinely collected in clinical practice; spirometry (most recent FEV1 and FVC) and medical history, e.g., admission history, comorbidities, medications, and smoking status. The anthropometric parameters included weight and BMI.

### 2.4. Intervention Dosing 

The intervention comprised two SunGold kiwifruit/day (provided by Zespri International, Mount Maunganui, New Zealand). SunGold kiwifruit comprise ~300 mg of vitamin C per two skinless fruit [22]. These were kept in cold storage and the participants were provided with punnets of fruit sufficient for two weeks at a time. The participants were asked to consume one in the morning and one in the evening. Participants were asked not to consume kiwifruit for the duration of the study unless provided as part of the study. Following the initiation of the kiwifruit intervention, one participant withdrew at two weeks due to illness/suspected kiwifruit allergy.

### 2.5. Dietary Intake Assessment

Recent dietary intakes were monitored using 24 h dietary recalls and were collected from participants at baseline and at each follow-up clinic visit (two weeks and six weeks). These were used to estimate fruit intake (150 g serves), vegetable intake (75 g serves), and dietary intakes of vitamin C (mg/d) using FoodWorks.online (Xyris, Brisbane, Australia). The recommended daily fruit and vegetable intakes in New Zealand are two servings of fruit and five to six servings of vegetables for females and males, respectively [23], and the recommended dietary intake for vitamin C is 45 mg/day [24]. Two kiwifruit are considered one serving of fruit [23].

### 2.6. Biosample Collection

Fasting blood samples (lithium heparin tube) and spot urine samples were collected from the participants at baseline and at each follow-up visit (two weeks and six weeks). Samples were processed and analysed for the different biomarkers as described below. The urine pottles and some of the plasma cryotubes contained the antioxidant butylatedhydroxytoluene (BHT) as a preservative for the oxidative stress biomarkers.

### 2.7. Vitamin C Analyses

Plasma vitamin C concentrations were assessed using high-performance liquid chromatography (HPLC) as described previously [25]. Plasma samples were processed immediately to stabilise the vitamin C in the plasma using metaphosphoric acid (10% *w*/*v*) containing the metal chelator diethylenetriamine pentaacetate (DTPA; 100 µmol/L); the supernatants were subsequently stored at −80 °C for batch analysis. Urine samples were processed similarly to plasma samples. Leukocytes were isolated from the remaining blood as described previously [26], and the vitamin C content of the leukocytes was analysed by HPLC [25]. 

### 2.8. Inflammatory Biomarker Analyses

Complete blood cell counts and differentials were carried out by Canterbury Health Laboratories, an International Accreditation NZ (IANZ) laboratory. The acute phase protein C-reactive protein was assessed by Canterbury Health Laboratories using endpoint nephelometry. The inflammatory cytokines interleukin-6 (IL-6) and tumour necrosis factor-α (TNF-α) were measured using enzyme-linked immunosorbent assays (ELISAs) according to the manufacturer’s instructions (ELISA MAXTM Deluxe Set, BioLegend, San Diego, CA, USA). 

### 2.9. Oxidative Biomarker Analyses

Lipid oxidation was assessed by measuring the gold standard marker F_2_-isoprostanes in plasma and urine samples using competitive ELISA according to the manufacturer’s instructions (Stat-8 Isoprostane ELISA kit, Cayman Chemical, Ann Arbor, MI, USA). Protein oxidation, i.e., protein carbonyls, was measured in plasma samples using sandwich ELISA as described previously [27].

### 2.10. Self-Reported Fatigue and Subjective Mood

Fatigue was assessed using the Multidimensional Fatigue Symptom Inventory-short form (MFSI-SF), which assesses general, physical, emotional, and mental fatigue, and vigour; a total fatigue score is derived from these [28]. Depression and anxiety were assessed using the Hospital Anxiety and Depression Scale (HADS) [29]. Scores of 0–7 are considered normal, 8–10 are mild, 11–15 are moderate, and 16–21 are severe.

### 2.11. Susceptibility and Severity of Respiratory Symptoms 

Participants received an automated daily text or email reminder to monitor respiratory infection symptoms during their six-week intervention period. If respiratory symptoms were present, the Wisconsin Upper Respiratory Symptom Survey-21 (WURSS-21) Daily Symptom Report was completed by the participants (as an online survey) to determine the severity and duration of the symptoms. After a four-week washout period, the participants were sent a daily reminder for another six-week period when no kiwifruit were being consumed as a comparator. 

### 2.12. Data Analysis 

Continuous data are presented as median (and interquartile range) or mean (and standard error of the mean), and categorical data as number (and percent), as indicated. The proportion of participants achieving adequate vitamin C status was calculated with binomial 95% confidence intervals (95%CI). Data were stratified by baseline characteristics. Between group comparisons were carried out using non-parametric Mann–Whitney U tests and linear correlations using Pearson coefficient and non-parametric correlations using Spearman coefficient, with *p* < 0.05 signifying statistically significant differences. Linear mixed effects models were used for time course data with Tukey’s post hoc analyses to correct for multiple comparisons. Statistical analyses and graphical outputs were generated using GraphPad Prism 9 (GraphPad, San Diego, CA, USA).

## 3. Results

### 3.1. Participant Characteristics 

We undertook two rounds of recruitment of community-dwelling individuals who had previously been hospitalised and received IV antimicrobials for severe respiratory exacerbations. The total cohort (*n* = 20) was predominantly female (65%) and of NZ European ethnicity (85%), with 3 (15%) identifying as Māori (Table 1). The median (IQR) age was 71 (67, 79) years (age range 31–84 years). The NZ deprivation index ranged from 1 to 10 with a median of 5 (2.3, 6.8). Most were non-smokers (only 1 was a current smoker). However, 12 (60%) had a history of pneumonia, and all had chronic respiratory-related conditions (bronchiectasis and/or COPD; Table 1). The median weight of the cohort was 65 (59, 78) kg (with a range of 44–105 kg), and the median BMI was 25 (21, 29) kg/m^2^ (with a range of 18–43 kg/m^2^). The cohort had a FEV1 of 1.5 (1.0, 1.9) L and a FVC of 2.4 (2.0, 3.1) L, with 15 (75%) having a FEV1/FVC ratio of ≤0.7 indicating obstructed lung function (Table 1). Two-thirds of the cohort had recent hospital visits for respiratory exacerbations at least once per year, and the median duration of IV antimicrobial therapy was 14 (5, 14) days (with a range of 2–21 days).

The recent dietary intake of the participants was determined using 24 h dietary recalls. Fruit and vegetable intake was found to be relatively low at a median (IQR) of 2.3 (0.6, 5.9) serves per day. Median vitamin C intakes were correspondingly low at 46 (5, 149) mg/day. Overall, 50% of the participants consumed less than the NZ recommended dietary intake (RDI) of 45 mg/day, and a large proportion (30%) had vitamin C intakes <10 mg/day, which is the minimum amount to prevent scurvy. There was no difference in vitamin C intake or fruit and vegetable intake between females and males (*p* > 0.05). 

### 3.2. Effect of Kiwifruit Intervention on Vitamin C Status

**Figure 1:**
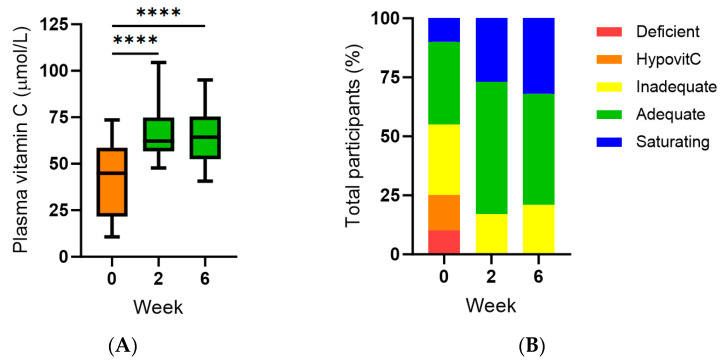

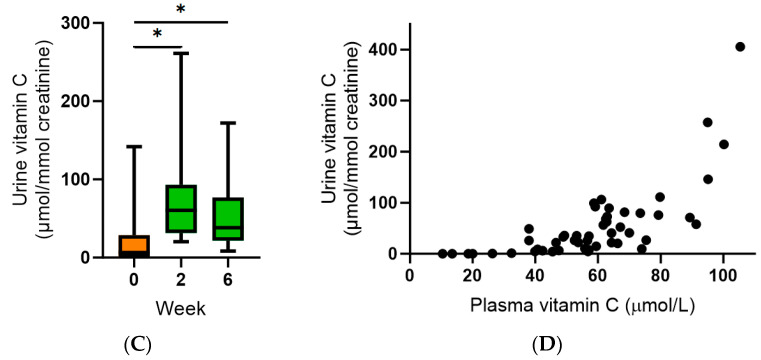
Effect of kiwifruit intervention on vitamin C status of the participants. (**A**) Plasma vitamin C concentrations of total cohort (*n* = 20); (**B**) plasma vitamin C was categorised as deficient (red; ≤11 µmol/L), hypovitaminosis C (orange; ≤23 µmol/L), inadequate (yellow; <50 µmol/L), adequate (green; ≥50 µmol/L), and saturating (blue; ≥70 µmol/L); (**C**) urinary vitamin C concentrations over time; (**D**) correlation of plasma and urinary vitamin C (r = 0.78, *p* < 0.0001). Box plots represent the median with borders being 25 and 75 percentiles and error bars being 10 and 90 percentiles; **** *p* < 0.0001, * *p* ≤ 0.05.

The median (IQR) plasma vitamin C status of the cohort at baseline was 45 (22, 59) µmol/L (Figure 1A). Of these, 55% (95%CI 34–74%) had inadequate vitamin C status (<50 µmol/L), 25% (95%CI 11–47%) had hypovitaminosis C (≤23 µmol/L), and 10% (95%CI 3–30%) had outright deficiency (≤11 µmol/L; Figure 1B). There was no difference in vitamin C status between males (*n* = 7) and females (42 [20, 53] µmol/L vs. 56 [22, 67] µmol/L; *p* > 0.05). Following intervention with two SunGold kiwifruit per day (equivalent to ~300 mg/day of vitamin C), there was a significant increase in plasma vitamin C concentrations to >60 µmol/L within two weeks, and these were maintained at six weeks (*p* < 0.0001; Figure 1A). All participants had concentrations above the hypovitaminosis standard, and there was an increase in the proportion of participants with adequate vitamin C status (79–83%; Figure 1B). The achievement of saturating vitamin C status was confirmed by increased urinary excretion of the vitamin following intervention (*p* = 0.02; Figure 1C), which occurs when circulating vitamin C reaches saturating concentrations and spills over into the urine (Figure 1D). Nevertheless, saturating plasma status was not achieved in more than two-thirds of the participants following 6 weeks of supplementation with two kiwifruit per day, and 21% (95%CI 8–43%) of the total cohort were unable to attain adequate vitamin C status (Figure 1B). Although leukocyte vitamin C concentrations were trending towards a significant association with plasma vitamin C status at baseline (r = 0.431, *p* = 0.058), there was no significant change in leukocyte vitamin C concentrations following intervention (*p* > 0.05), likely due to the median vitamin C concentrations being already relatively high at baseline (1.1 [1.0, 2.2] nmol/10^6^ cells).

**Figure 2:**
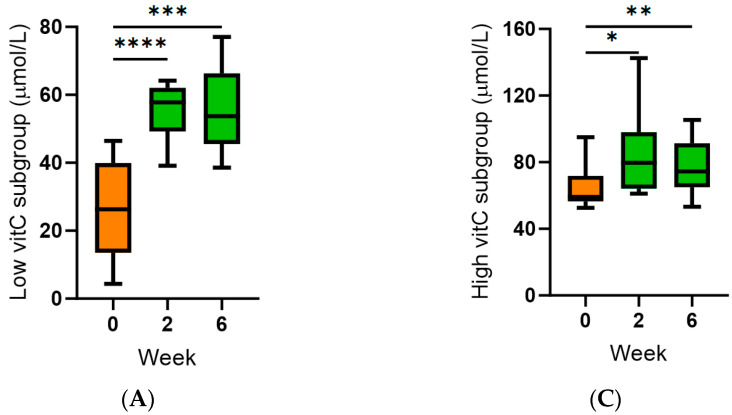

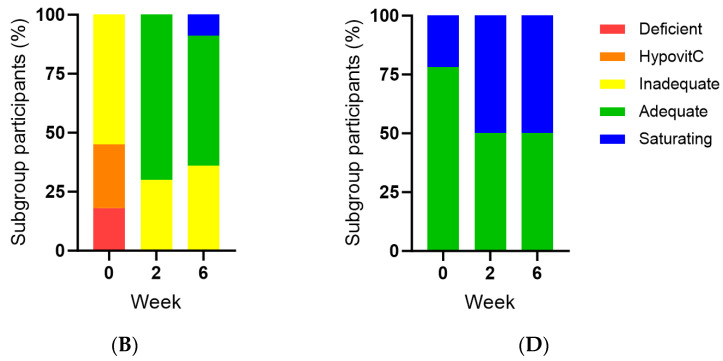
Effect of kiwifruit intervention on low and high vitamin C subgroups. (**A**,**B**) Subgroup with inadequate baseline vitamin C concentrations (*n* = 11, <50 µmol/L); (**C**,**D**) subgroup with adequate baseline vitamin C concentrations (*n* = 9, ≥50 µmol/L). Plasma vitamin C was categorised as deficient (red; ≤11 µmol/L), hypovitaminosis C (orange; ≤23 µmol/L), inadequate (yellow; <50 µmol/L), adequate (green; ≥50 µmol/L), and saturating (blue; ≥70 µmol/L). Box plots represent the median with borders being 25 and 75 percentiles and error bars being 10 and 90 percentiles; **** *p* < 0.0001, *** *p* < 0.001, ** *p* < 0.01, * *p* < 0.05.

Subgroup analyses indicated that it was the group of participants with inadequate (<50 µmol/L) baseline vitamin C concentrations (*n* = 11; 26 [14, 40] µmol/L) who benefitted the most from supplementation (Figure 2A,B) relative to those who already had adequate (≥50 µmol/L) vitamin C status at baseline (*n* = 9; 59 [57, 72] µmol/L; Figure 2C,D). One-third of the low vitamin C subgroup were unable to reach adequate plasma concentrations following kiwifruit intervention (Figure 2B). 

**Figure 3:**
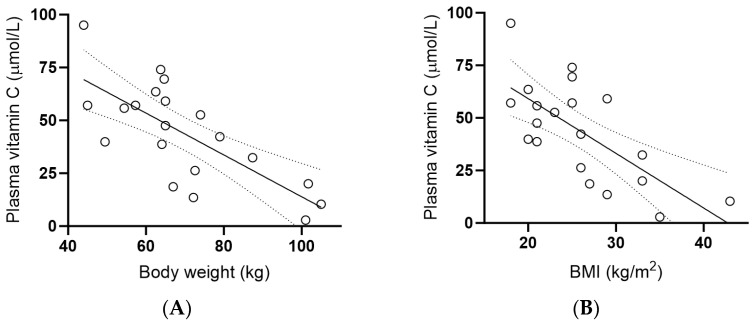
Correlations between plasma vitamin C status and body weight or BMI. (**A**) Inverse correlation between body weight and baseline plasma vitamin C (r = −0.734, *p* = 0.0002). (**B**) Inverse correlation between BMI and plasma vitamin C (r = −0.696, *p* = 0.0007).

There were no differences in the baseline vitamin C intakes between the lower and higher plasma vitamin C subgroups (*p* > 0.05), suggesting factors other than dietary intake were impacting baseline vitamin C status. One of the study participants was a current smoker, which is known to enhance vitamin C turnover; they had hypovitaminosis C at baseline (i.e., <23 µmol/L) and were unable to achieve >50 µmol/L following six-week supplementation with two kiwifruit per day. There was also a strong inverse correlation between baseline plasma vitamin C status and body weight (r = −0.734, *p* = 0.0002) and BMI (r = −0.696, *p* = 0.0007; Figure 3A,B). This was not due to lower vitamin C intake in people of higher body weight, as there was no association between weight or BMI and vitamin C intake (*p* > 0.05). Overall, the lower vitamin C group (<50 µmol/L) had higher weight and BMI than the higher vitamin C group, i.e., 73 (65, 101) kg vs. 63 (50, 65) kg (*p* = 0.006) and 27 (21, 33) kg/m^2^ vs. 23 (19, 25) kg/m^2^ (*p* = 0.03). Two of the three study participants with body weight >100 kg, who also had hypovitaminosis C or outright deficiency at baseline, were unable to achieve adequate vitamin C status following six weeks of kiwifruit supplementation.

### 3.3. Effect of Kiwifruit Intervention on Inflammatory and Oxidative Biomarkers

**Figure 4:**
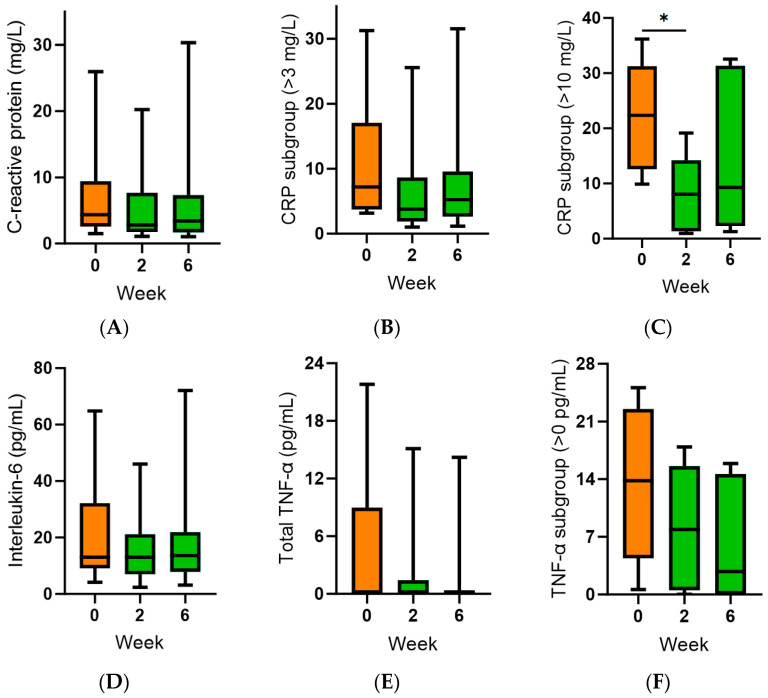
Effect of kiwifruit intervention on inflammatory biomarkers over time. (**A**) Total plasma C-reactive protein concentrations; (**B**) subgroup with baseline C-reactive protein (CRP) >3 mg/L (*n* = 14); (**C**) subgroup with baseline C-reactive protein (CRP) >10 mg/L (*n* = 5); (**D**) interleukin-6 concentrations; (**E**) total TNF-α concentrations; (**F**) subgroup with baseline TNF-α concentrations >0 pg/mL (*n* = 7). Box plots represent the median with borders being 25 and 75 percentiles and error bars being 10 and 90 percentiles; * *p* < 0.05.

The inflammatory biomarker C-reactive protein (CRP) was elevated at baseline (4.4 [2.3, 9.4] mg/L). This did not change following kiwifruit intervention (*p* > 0.05; Figure 4A). Subgroup analysis of the participants with baseline CRP concentrations >3 mg/L (*n* = 14) showed a decrease in median CRP following the intervention, which did not reach statistical significance (*p* > 0.05; Figure 4B). Further subgroup analysis of participants with baseline CRP > 10 mg/L (*n* = 5) showed a significant decrease at week 2 (*p* = 0.02), but this was not maintained at week 6 (*p* > 0.05; Figure 4C). Baseline interleukin 6 (IL-6) concentrations were 13 (9, 32) pg/mL, and these correlated closely with CRP concentrations (r = 0.724, *p* = 0.0003). There was no effect of intervention on IL-6 concentrations over time (*p* > 0.05; Figure 4D). Baseline tumour necrosis factor-α (TNF-α) concentrations were low (0 [0, 9] pg/mL) because only 7 (35%) of the cohort had values greater than zero (14 [4, 23] pg/mL). Kiwifruit intervention had no statistically significant effect on TNF-α concentrations over time (*p* > 0.05; Figure 4E), although median values were trending downwards in the high TNF-α subgroup (*n* = 7, *p* > 0.05; Figure 4F). Inverse correlations observed between plasma vitamin C status and the inflammatory biomarkers were weak (e.g., r = −0.18 for CRP) and did not reach statistical significance (*p* > 0.05).

Urinary F_2_isoprostanes, a marker of lipid oxidation, were elevated at 1.6 (1.0, 3.2) ng/mg creatinine, comparable to concentrations we have previously observed in smokers. However, there was no change in these concentrations following six weeks of kiwifruit intervention (*p* > 0.05). Plasma F_2_isoprostane concentrations were 0.1 (0.04, 0.25) ng/mL at baseline and did not change following intervention (*p* > 0.05). Protein carbonyls, a marker of protein oxidation, were 0.25 (0.21, 0.27) nmol/mg protein at baseline. These concentrations were comparable to those we have previously observed in healthy controls and, unsurprisingly, did not change following intervention (*p* > 0.05). 

### 3.4. Effect of Kiwifruit Intervention on Self-Reported Fatigue and Subjective Mood

**Figure 5:**
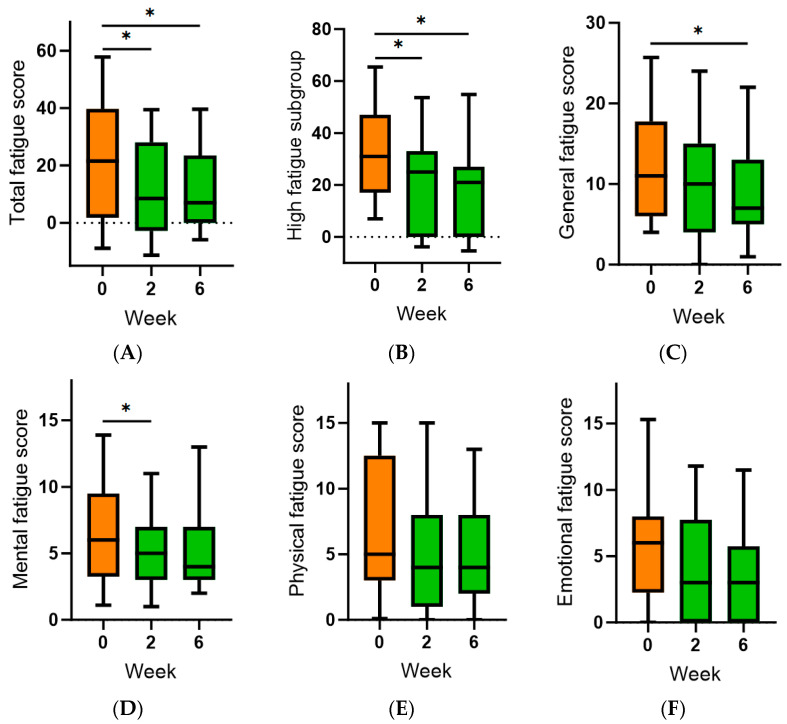
Effect of kiwifruit intervention on self-reported fatigue over time. (**A**) Total fatigue; (**B**) total fatigue subgroup (*n* = 15 with baseline score > 0); (**C**) general fatigue subscale; (**D**) mental fatigue subscale; (**E**) physical fatigue subscale; (**F**) emotional fatigue subscale. Total fatigue is the sum of general, mental, physical and emotional fatigue minus the vigour score. Box plots represent the median with borders being 25 and 75 percentiles and error bars being 10 and 90 percentiles; * *p* < 0.05.

Self-reported fatigue was assessed using the Multi-dimensional Fatigue Symptom Inventory (MFSI). The total fatigue score (which is the sum of general, mental, physical and emotional fatigue, minus the vigour score) was 22 (1.8, 40) at baseline. There were significant decreases in total fatigue at both weeks two and six following kiwifruit intervention (*p* < 0.05; Figure 5A). This was even more apparent when looking at the subgroup that reported fatigue scores >0 at baseline (*n* = 15; Figure 5B). This appeared to be primarily due to decreases in the general and mental fatigue subscales (Figure 5C,D). There were no effects of kiwifruit intervention on the physical and emotional fatigue subscales (*p* > 0.05; Figure 5E,F). Inverse correlations observed between plasma vitamin C status and fatigue scores were weak (e.g., r = −0.15 for general and emotional fatigue) and did not reach statistical significance (*p* > 0.05).

**Figure 6:**
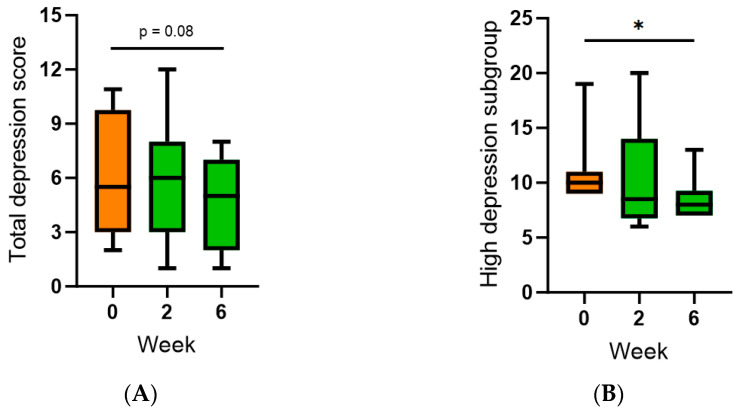
Effect of kiwifruit intervention on subjective mood over time. (**A**) Total depression; (**B**) high depression subgroup (*n* = 7 with baseline score ≥ 8). Box plots represent the median with borders being 25 and 75 percentiles and error bars being 10 and 90 percentiles; * *p* < 0.05.

The participant’s subjective mood was monitored using the Hospital Anxiety and Depression Scale (HADS). The baseline anxiety score was 7 (3, 9), and the baseline depression score was 6 (3, 10); the normal range is a score of ≤7. At baseline, 45% of the cohort had mild to severe anxiety, and 35% of the cohort had mild to severe depression (i.e., score of ≥8). Following intervention with SunGold kiwifruit, there was no change in anxiety in the total cohort (*p* > 0.05) or the higher anxiety subgroup (*n* = 9; *p* > 0.05). However, there was a trend towards a decrease in depression in the whole cohort (*p* = 0.08, Figure 6A) and a significant decrease at week six in the higher depression subgroup (*n* = 7; *p* = 0.03, Figure 6B).

### 3.5. Effect of Kiwifruit on Respiratory Symptom Incidence, Duration and Severity

The incidence, duration, and severity of respiratory symptoms were monitored using the Wisconsin Upper Respiratory Symptom Survey (WURSS). The participants completed the WURSS daily for the six weeks of the kiwifruit intervention if they had respiratory symptoms then undertook a four-week washout period, followed by another six weeks of completing a daily WURSS. Although there was no difference in the total number of participants reporting symptoms in the first phase (10 of 19; 53% [95%CI 32–73%]) relative to the second phase (11 of 18; 61% [95%CI 39–80%]), there was a lower proportion of individual symptoms reported in the first phase (8.5 [7.5, 9.3]) relative to the second phase (10 [8.8, 10]; *p* = 0.05; Table 2). As such, kiwifruit intervention appeared to decrease the number of different symptoms reported. Although the total and median duration of symptoms appeared shorter in the first phase relative to the second phase, these did not reach statistical significance (*p* > 0.05; Table 2). There were no differences in the total or median severity of the symptoms (*p* > 0.05; Table 2), and there were also no differences in how sick the participants felt overall between the two phases; 2.9 (2.2, 3.1) vs. 2.3 (1.5, 3.0), with 1 indicating feeling very mildly sick and 3 indicating feeling mildly sick.

## 4. Discussion

In this study, people with a history of severe respiratory infections received two SunGold kiwifruit per day for six weeks as a supplement to their normal diet to determine if circulating concentrations of immune-supportive vitamin C were increased at follow-up. At baseline, the study participants had a relatively low recent fruit and vegetable intake of 2.3 servings per day, the NZ recommendations being 7–8 servings per day [23]. Correspondingly, their recent dietary intake of vitamin C was also low at 46 mg/day; the median NZ vitamin C intake was 99 mg/day (in 2008/2009) [31]. Of these, a large proportion (30%) had vitamin C intakes <10 mg/day, the minimum amount to prevent scurvy, and 50% of the participants consumed less than the NZ recommended dietary intake (RDI) of 45 mg/day [31]. The median baseline circulating vitamin C concentration was 45 µmol/L, and 55% of the participants had inadequate vitamin C status (<50 µmol/L), 25% had hypovitaminosis C (≤23 µmol/L), and 10% had outright deficiency (≤11 µmol/L). This is a higher proportion of hypovitaminosis C and deficiency than has been previously reported in the same general population [32].

Following intervention with two SunGold kiwifruit per day (equivalent to ~300 mg/day of vitamin), there was a significant increase in plasma vitamin C concentrations to greater than 60 µmol/L within two weeks, and these were maintained at six weeks. This corresponded to a complete decrease in participants with hypovitaminosis C and deficiency and an increase in participants with adequate and saturating vitamin C status (~80%). Subgroup analyses indicated that it was the group of participants with inadequate baseline vitamin C concentrations (<50 µmol/L) who benefitted the most from supplementation relative to those who already had adequate vitamin C status at baseline. Nevertheless, >20% of the study participants were unable to achieve adequate vitamin C status following supplementation with two kiwifruit per day. 

Previous research has shown that certain vulnerable subgroups within the population have higher vitamin C requirements, such as smokers and those with higher body weight [33]. One of the study participants who were unable to achieve >50 µmol/L following six weeks of kiwifruit supplementation was a current smoker, which is known to enhance vitamin C turnover. There was also a strong inverse correlation between baseline plasma vitamin C status and body weight and BMI in the study participants. This suggests that people who have higher body weight (or who smoke) either need a larger dose of kiwifruit (or other vitamin C-rich foods) and/or a longer intervention period to allow for full repletion of body stores. Due to the ongoing obesity pandemic, both in NZ and globally [34], a dose-finding study in people of higher body weight is indicated to determine the optimal fruit intake to reach optimal vitamin C status. 

The association between body mass and respiratory infection, including COVID-19, is complex, with both overweight and underweight individuals at increased risk [35,36,37,38]. As such, optimising vitamin C status via higher dietary intake may help to support important immune and other physiological functions [1,39]. Hunter et al. [20] previously reported improvements in the severity and duration of selected respiratory symptoms in older adults supplemented with the equivalent of four kiwifruit (fresh and dried). Although not powered to detect effects on respiratory symptoms, using the daily Wisconsin Upper Respiratory Symptom Survey (WURSS), we did observe a slightly lower proportion of individual respiratory symptoms in the kiwifruit intervention phase of the study relative to an equivalent phase when no kiwifruit was consumed (8.5 vs. 10, respectively). Although we did not confirm a shorter duration of symptoms during the kiwifruit intervention phase, a clinically meaningful difference remains possible as the point estimate of symptom duration was lower, and the large variability between participants meant our study was not powered to detect a difference. Rolling recruitment meant the participants completed their WURSS at different times throughout a 9-month period, partially accounting for seasonal effects on respiratory symptoms.

Following the kiwifruit intervention, we also observed small improvements in participants’ self-reported fatigue and subjective mood, specifically depression. We previously showed, using the Profile of Mood States (POMS) questionnaire, that the supplementation of young adult males with two Gold kiwifruit per day for six weeks was able to improve total mood disturbance score, decrease fatigue and trended towards decreased depression in the participants who had high mood disturbance at baseline [40]. Others have shown comparable effects of kiwifruit intervention on mood and vitality [41]. In the current study, we used the Multidimensional Fatigue Symptom Inventory (MFSI) and Hospital Anxiety and Depression Scale (HADS) to assess fatigue and subjective mood, respectively. The observation of decreased fatigue and depression following kiwifruit supplementation in different cohorts assessed using different questionnaires supports the veracity of the findings [42]. Although vitamin C has numerous mood-enhancing properties, kiwifruit comprise other constituents which can also support beneficial mood [40]. As such, the mechanisms of action are not certain, although indications towards decreased systemic inflammatory biomarker concentrations were observed in the current study (e.g., CRP and TNF-α), particularly in the subgroups with elevated baseline biomarkers. However, the non-specific nature of circulating inflammatory biomarkers precludes understanding what may be occurring within the tissues of the central nervous system itself.

A strength of the study includes providing vitamin C in the form of whole food, which maximises health benefits via the concurrent provision of other healthy plant constituents [43]; we previously showed no effect of the food matrix on the uptake of vitamin C [19]. A limitation of a whole-food intervention is the inability to discern which constituent(s) may be causing the observed effects, particularly since some constituents, such as vitamins C and E, can act synergistically [44]. Another limitation was the pre-post-study design, which, unlike an RCT, does not allow for complete certainty that the changes in outcomes are a result of the intervention or other changes made by participants. It should be noted, however, that it is very difficult to have proper control groups in nutrient intervention studies as all participants are consuming variable amounts of the nutrient via their daily diet [45]. The participants were not pre-screened for low baseline vitamin C status, which is another limitation, as beneficial effects are more likely to be seen in those with inadequate vitamin status at baseline [46]. Although the study was appropriately powered to detect changes in the vitamin C status of the participants, a limitation was the large variability in the other outcomes assessed (i.e., inflammatory biomarkers, fatigue and subjective mood, and respiratory symptoms). Since there were indications of improvements in some of these parameters, larger fully powered studies with a control group to account for the Hawthorn effect appear warranted, particularly studies that pre-screen for baseline dysregulation. Furthermore, because obesity tends to be associated with elevated inflammation, increased fatigue and depression, and increased susceptibility to respiratory infections, restoring adequate to optimal vitamin C status in people who are overweight/have obesity may help to decrease these symptoms. As such, future clinical trials could pre-screen for both lower vitamin C status and higher body weight at baseline to test these premises.

## 5. Conclusions

A relatively large proportion of the study participants presented with hypovitaminosis C and outright deficiency even when not undergoing a current respiratory exacerbation. This indicates that vitamin C insufficiency is present in these people, even in the absence of acute infection. Supplementation with two SunGold kiwifruit per day for six weeks was able to restore adequate to saturating vitamin C concentrations in ~80% of the study participants. Smoking and larger body weight likely contributed to the ~20% of participants who were unable to achieve adequate vitamin C status. Thus, smokers and people with higher body weight may need higher doses of kiwifruit and/or a longer duration of supplementation to help replete depleted body stores. The contribution of vitamin C to reducing fatigue, depression, and number of respiratory symptoms warrants further investigation.

## Figures and Tables

**Table 1:** Participant demographic and anthropometric characteristics.

Parameter N (%)	Total Cohort (*n* = 20)	Variable Median (IQR)	Total Cohort (*n* = 20)
Sex:		Age, years	71 (67, 79)
Female	13 (65)	NZDep index	5 (2.3, 6.8)
Male	7 (35)	Weight, kg	65 (59, 78)
Ethnicity:		BMI, kg/m^2^	25 (21, 29)
NZ/European	18 (90)	FEV1, L	1.5 (1.0, 1.9)
Māori	3 (15)	% predicted	61 (46, 90)
Smoking status:		FVC, L	2.4 (2.0, 3.1)
Never smoked	17 (85)	% predicted	91 (73, 103)
Former smoker	2 (10)	FEV1/FVC ratio	0.6 (0.4, 0.8)
Current smoker	1 (5)	Recent hospital visits, %	
Respiratory conditions:		<1/y:~1/y:>1/y IV Ab duration, days	35:40:25 14 (5, 14)
History of pneumonia Bronchiectasis COPD	12 (60) 19 (95) 6 (30)		
Comorbidities:			
Coronary artery disease Asthma Depression	6 (30) 6 (30) 3 (15)		

BMI, body mass index; COPD, chronic obstructive pulmonary disease; FEV1, forced expiratory volume in 1 s; FVC, forced vital capacity; IV Ab, intravenous antibiotics/antimicrobials; NZDep, NZ Deprivation 2018. Normal spirometry findings are FEV1/FVC ratio > 0.70 and both FEV1 and FVC > 80% of predicted value [30].

**Table 2:** Effect of intervention on respiratory symptom incidence, duration, and severity.

	Weeks 1–6 (2 Kiwifruit/Day)			Weeks 11–16 (No Kiwifruit)		
Symptom	Number of Participants	Duration of Symptoms	Severity of Symptoms	Number of Participants	Duration of Symptoms	Severity of Symptoms
Individual symptoms:						
Runny nose	9	3.0 (2.0, 16)	2.3 (2.2, 3.5)	9	6.0 (4.0, 12)	2.5 (1.3, 3.0)
Plugged nose	6	3.0 (2.0, 7.5)	1.4 (1.0, 2.6)	8	5.5 (3.3, 9.5)	2.7 (1.3, 2.8)
Sneezing	8	5.0 (2.0, 13)	2.2 (1.2, 2.8)	9	6.0 (3.0, 10)	1.7 (1.4, 2.5)
Sore throat	8	4.5 (1.3, 11)	1.8 (1.0, 2.8)	10	4.5 (1.8, 9.5)	2.7 (2.0, 3.0)
Scratchy throat	8	5.5 (1.3, 11)	1.9 (1.2, 3.9)	10	4.0 (2.8, 8.0)	2.3 (2.0, 2.5)
Cough	10	4.5 (2.0, 21)	4.3 (2.5, 5.3)	11	10 (3.0, 11)	2.7 (1.8, 3.0) *
Hoarseness	6	9.0 (2.0, 23)	3.4 (2.6, 5.1)	10	6.0 (2.8, 11)	2.7 (1.7, 2.9)
Head congestion	9	6.0 (2.0, 18)	2.8 (1.7, 3.9)	8	8.0 (5.5, 11)	2.2 (1.5, 2.7)
Chest congestion	9	4.0 (2.0, 16)	4.0 (2.7, 5.7)	10	7.5 (3.8, 11)	2.6 (2.2, 3.3)
Feeling tired	10	4.5 (2.0, 20)	3.5 (2.2, 6.1)	11	9.0 (3.0, 12)	3.0 (2.3, 4.0)
Median symptoms	8.5 (7.5, 9.3)	3.3 (1.8, 14)	3.0 (1.7, 3.8)	10 (8.8, 10) *	7.3 (3.0, 10)	2.4 (2.1, 2.8)
Total symptoms	10 of 19	5.0 (2.8, 22)	21 (10, 33)	11 of 18	10 (4.0, 12)	21 (10, 22)

Data represent the median (Q1, Q3). Weeks 7–10 were the washout period. * *p* ≤ 0.05.

## Data Availability

Data described in the manuscript will be made available upon reasonable request pending application and approval.
